# 4-(Diphenyl­amino)benzaldehyde

**DOI:** 10.1107/S1600536808042311

**Published:** 2008-12-17

**Authors:** Hongli Wang, Wenyuan Xu, Bin Zhang, Wenjing Xiao, Hong Wu

**Affiliations:** a Department of Chemistry, Central China Normal University, Wuhan, Hubei 430079, People’s Republic of China

## Abstract

In the title compound, C_19_H_15_NO, the N atom adopts an approximately trigonal-planar geometry, lying 0.07 (1) Å from the plane defined by its three neighbouring C atoms. The two phenyl rings and the benzaldehyde group form dihedral angles of 53.0 (1)/47.2 (1) and 29.0 (1)°, respectively, with this central plane.

## Related literature

For details of the synthesis, see: Wang & Zhou (2000 ▶). For aryl­amines, see: Beller (1995 ▶); Wang *et al.* (2005 ▶); Yao *et al.* (2006 ▶).
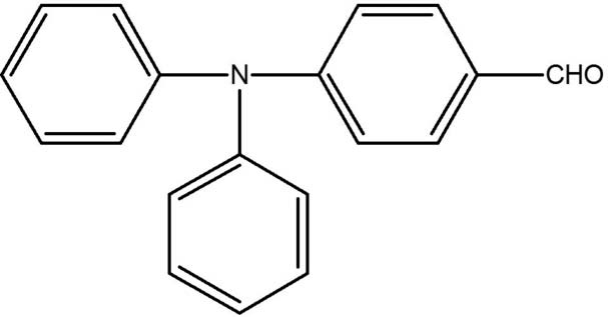


## Experimental

### 

#### Crystal data

C_19_H_15_NO
*M* *_r_* = 273.32
Monoclinic, 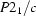

*a* = 12.1188 (8) Å
*b* = 11.4342 (8) Å
*c* = 10.9560 (7) Å
β = 102.082 (2)°
*V* = 1484.53 (17) Å^3^
*Z* = 4
Mo *K*α radiation
μ = 0.08 mm^−1^
*T* = 292 (2) K
0.40 × 0.10 × 0.04 mm

#### Data collection

Bruker SMART CCD area-detector diffractometer
Absorption correction: multi-scan (*SADABS*; Bruker, 2000 ▶) *T*_min_ = 0.971, *T*_max_ = 0.997
12673 measured reflections
2898 independent reflections
1393 reflections with *I* > 2σ(*I*)
*R*_int_ = 0.087

#### Refinement

*R*[*F* ^2^ > 2σ(*F* ^2^)] = 0.056
*wR*(*F* ^2^) = 0.153
*S* = 0.91
2898 reflections
191 parameters
H-atom parameters constrained
Δρ_max_ = 0.14 e Å^−3^
Δρ_min_ = −0.14 e Å^−3^

### 

Data collection: *SMART* (Bruker, 2000 ▶); cell refinement: *SAINT* (Bruker, 2000 ▶); data reduction: *SAINT*; program(s) used to solve structure: *SHELXS97* (Sheldrick, 2008 ▶); program(s) used to refine structure: *SHELXL97* (Sheldrick, 2008 ▶); molecular graphics: *SHELXTL* (Sheldrick, 2008 ▶); software used to prepare material for publication: *SHELXTL*.

## Supplementary Material

Crystal structure: contains datablocks global, I. DOI: 10.1107/S1600536808042311/bi2326sup1.cif

Structure factors: contains datablocks I. DOI: 10.1107/S1600536808042311/bi2326Isup2.hkl

Additional supplementary materials:  crystallographic information; 3D view; checkCIF report

## Figures and Tables

**Fig. 1.**
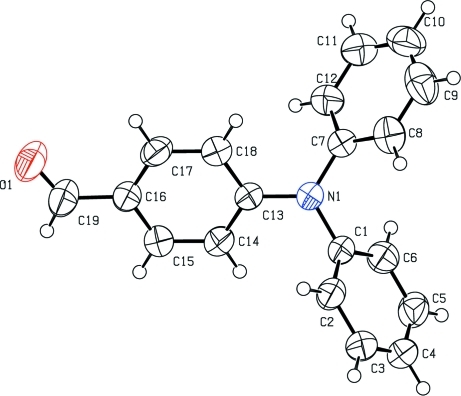
Molecular structure of the title compound showing displacement ellipsoids at 50% probability for non-H atoms.

## supplementary crystallographic information

### Comment

Arylamine derivatives are common intermediates in the synthesis of many compounds and polymers (Yao *et al.*, 2006; Beller, 1995). We became interested in using the Vilsmeier reaction to obtain the title compound, which is a good intermediate for several compounds (Wang *et al.*, 2005). In the crystal structure (Fig. 1), the bond lengths and angles are within normal ranges.

### Experimental

The title compound was synthesised according to the published procedure (Wang & Zhou, 2000) and recrystallized from chloroform.

### Refinement

All H atoms were placed in geometrically idealized positions with C—H = 0.93 Å and refined using a riding model, with *U*_iso_(H) = 1.2*U*_eq_(C).

### Crystal data

**Table:**

C_19_H_15_NO	*F*(000) = 576
*M**_r_* = 273.32	*D*_x_ = 1.223 Mg m^−^^3^
Monoclinic, *P*2_1_/*c*	Mo *K*α radiation, λ = 0.71073 Å
Hall symbol: -P 2ybc	Cell parameters from 843 reflections
*a* = 12.1188 (8) Å	θ = 2.6–18.1°
*b* = 11.4342 (8) Å	µ = 0.08 mm^−^^1^
*c* = 10.9560 (7) Å	*T* = 292 K
β = 102.082 (2)°	Needle, colorless
*V* = 1484.53 (17) Å^3^	0.40 × 0.10 × 0.04 mm
*Z* = 4	

### Data collection

**Table:**

Bruker SMART CCD area-detector diffractometer	2898 independent reflections
Radiation source: fine-focus sealed tube	1393 reflections with *I* > 2σ(*I*)
graphite	*R*_int_ = 0.087
φ and ω scans	θ_max_ = 26.0°, θ_min_ = 1.7°
Absorption correction: multi-scan (*SADABS*; Bruker, 2000)	*h* = −14→14
*T*_min_ = 0.971, *T*_max_ = 0.997	*k* = −14→14
12673 measured reflections	*l* = −13→13

### Refinement

**Table:**

Refinement on *F*^2^	Secondary atom site location: difference Fourier map
Least-squares matrix: full	Hydrogen site location: inferred from neighbouring sites
*R*[*F*^2^ > 2σ(*F*^2^)] = 0.056	H-atom parameters constrained
*wR*(*F*^2^) = 0.153	*w* = 1/[σ^2^(*F*_o_^2^) + (0.0688*P*)^2^] where *P* = (*F*_o_^2^ + 2*F*_c_^2^)/3
*S* = 0.91	(Δ/σ)_max_ < 0.001
2898 reflections	Δρ_max_ = 0.14 e Å^−^^3^
191 parameters	Δρ_min_ = −0.13 e Å^−^^3^
0 restraints	Extinction correction: *SHELXL97* (Sheldrick, 2008), Fc^*^=kFc[1+0.001xFc^2^λ^3^/sin(2θ)]^-1/4^
Primary atom site location: structure-invariant direct methods	Extinction coefficient: 0.007 (2)

### Special details

**Table:**

Geometry. All e.s.d.'s (except the e.s.d. in the dihedral angle between two l.s. planes) are estimated using the full covariance matrix. The cell e.s.d.'s are taken into account individually in the estimation of e.s.d.'s in distances, angles and torsion angles; correlations between e.s.d.'s in cell parameters are only used when they are defined by crystal symmetry. An approximate (isotropic) treatment of cell e.s.d.'s is used for estimating e.s.d.'s involving l.s. planes.
Refinement. Refinement of *F*^2^ against ALL reflections. The weighted *R*-factor *wR* and goodness of fit *S* are based on *F*^2^, conventional *R*-factors *R* are based on *F*, with *F* set to zero for negative *F*^2^. The threshold expression of *F*^2^ > σ(*F*^2^) is used only for calculating *R*-factors(gt) *etc*. and is not relevant to the choice of reflections for refinement. *R*-factors based on *F*^2^ are statistically about twice as large as those based on *F*, and *R*- factors based on ALL data will be even larger.

### Fractional atomic coordinates and isotropic or equivalent isotropic displacement parameters (Å^2^)

**Table:**

	*x*	*y*	*z*	*U*_iso_*/*U*_eq_	
C1	0.7834 (2)	0.14287 (18)	0.0025 (2)	0.0483 (7)	
C2	0.6819 (2)	0.09360 (19)	0.0112 (3)	0.0587 (8)	
H2	0.6307	0.1360	0.0460	0.070*	
C3	0.6555 (2)	−0.0192 (2)	−0.0318 (3)	0.0624 (8)	
H3	0.5864	−0.0520	−0.0266	0.075*	
C4	0.7312 (3)	−0.0821 (2)	−0.0817 (3)	0.0637 (8)	
H4	0.7136	−0.1578	−0.1102	0.076*	
C5	0.8329 (3)	−0.0340 (2)	−0.0896 (3)	0.0659 (8)	
H5	0.8841	−0.0771	−0.1238	0.079*	
C6	0.8601 (2)	0.0793 (2)	−0.0468 (2)	0.0574 (7)	
H6	0.9295	0.1116	−0.0514	0.069*	
C7	0.9178 (2)	0.27936 (18)	0.1276 (2)	0.0492 (7)	
C8	0.9540 (2)	0.2101 (2)	0.2309 (3)	0.0645 (8)	
H8	0.9076	0.1514	0.2505	0.077*	
C9	1.0605 (3)	0.2283 (3)	0.3059 (3)	0.0787 (9)	
H9	1.0850	0.1817	0.3759	0.094*	
C10	1.1294 (3)	0.3147 (3)	0.2769 (3)	0.0790 (10)	
H10	1.2007	0.3263	0.3267	0.095*	
C11	1.0929 (3)	0.3832 (2)	0.1750 (3)	0.0811 (10)	
H11	1.1392	0.4421	0.1555	0.097*	
C12	0.9878 (2)	0.3656 (2)	0.1008 (3)	0.0639 (8)	
H12	0.9638	0.4128	0.0312	0.077*	
C13	0.7402 (2)	0.35282 (17)	−0.0019 (2)	0.0475 (6)	
C14	0.6713 (2)	0.34636 (19)	−0.1196 (3)	0.0593 (7)	
H14	0.6724	0.2797	−0.1679	0.071*	
C15	0.6009 (2)	0.43842 (19)	−0.1657 (3)	0.0612 (8)	
H15	0.5539	0.4320	−0.2442	0.073*	
C16	0.5989 (2)	0.53936 (19)	−0.0979 (3)	0.0556 (7)	
C17	0.6677 (2)	0.5467 (2)	0.0200 (3)	0.0605 (8)	
H17	0.6668	0.6142	0.0673	0.073*	
C18	0.7375 (2)	0.45501 (19)	0.0682 (3)	0.0577 (7)	
H18	0.7830	0.4610	0.1476	0.069*	
C19	0.5231 (2)	0.6343 (2)	−0.1488 (3)	0.0772 (9)	
H19	0.4757	0.6210	−0.2260	0.093*	
N1	0.81100 (18)	0.25939 (15)	0.0479 (2)	0.0567 (6)	
O1	0.51528 (17)	0.72793 (15)	−0.1018 (2)	0.0929 (8)	

### Atomic displacement parameters (Å^2^)

**Table:**

	*U*^11^	*U*^22^	*U*^33^	*U*^12^	*U*^13^	*U*^23^
C1	0.0461 (16)	0.0370 (12)	0.0592 (17)	0.0004 (11)	0.0050 (14)	0.0024 (11)
C2	0.0541 (17)	0.0434 (14)	0.078 (2)	0.0030 (12)	0.0132 (15)	−0.0002 (13)
C3	0.0542 (18)	0.0487 (15)	0.080 (2)	−0.0060 (13)	0.0049 (17)	0.0013 (13)
C4	0.070 (2)	0.0444 (14)	0.069 (2)	0.0000 (15)	−0.0034 (17)	−0.0046 (13)
C5	0.077 (2)	0.0587 (17)	0.0599 (19)	0.0171 (15)	0.0085 (17)	−0.0094 (13)
C6	0.0515 (17)	0.0579 (15)	0.0632 (19)	−0.0006 (13)	0.0126 (15)	0.0002 (13)
C7	0.0497 (17)	0.0421 (13)	0.0533 (17)	−0.0006 (12)	0.0052 (14)	−0.0027 (12)
C8	0.063 (2)	0.0678 (17)	0.063 (2)	0.0082 (14)	0.0146 (17)	0.0088 (15)
C9	0.077 (3)	0.102 (2)	0.054 (2)	0.030 (2)	0.0059 (19)	−0.0009 (17)
C10	0.059 (2)	0.091 (2)	0.080 (3)	0.0012 (19)	−0.001 (2)	−0.035 (2)
C11	0.068 (2)	0.0638 (18)	0.105 (3)	−0.0069 (16)	0.004 (2)	−0.0098 (19)
C12	0.0566 (19)	0.0577 (15)	0.073 (2)	−0.0066 (14)	0.0024 (16)	0.0013 (14)
C13	0.0466 (16)	0.0380 (12)	0.0558 (17)	−0.0024 (11)	0.0063 (14)	−0.0006 (11)
C14	0.0660 (19)	0.0441 (14)	0.0625 (19)	0.0027 (12)	0.0010 (16)	−0.0066 (12)
C15	0.0591 (18)	0.0515 (15)	0.0662 (19)	0.0039 (13)	−0.0025 (15)	0.0006 (13)
C16	0.0484 (17)	0.0430 (14)	0.073 (2)	0.0013 (11)	0.0066 (16)	0.0000 (13)
C17	0.0591 (18)	0.0417 (14)	0.081 (2)	−0.0023 (13)	0.0158 (17)	−0.0126 (13)
C18	0.0561 (18)	0.0489 (14)	0.0638 (19)	0.0003 (12)	0.0027 (15)	−0.0047 (13)
C19	0.077 (2)	0.0477 (16)	0.103 (3)	0.0085 (15)	0.0115 (19)	0.0079 (16)
N1	0.0526 (14)	0.0375 (10)	0.0714 (16)	0.0000 (9)	−0.0067 (12)	0.0017 (10)
O1	0.0878 (16)	0.0471 (11)	0.143 (2)	0.0126 (10)	0.0210 (15)	−0.0011 (11)

### Geometric parameters (Å, °)

**Table:**

C1—C2	1.375 (3)	C10—C11	1.359 (4)
C1—C6	1.376 (3)	C10—H10	0.930
C1—N1	1.437 (3)	C11—C12	1.375 (3)
C2—C3	1.387 (3)	C11—H11	0.930
C2—H2	0.930	C12—H12	0.930
C3—C4	1.366 (4)	C13—C14	1.383 (3)
C3—H3	0.930	C13—C18	1.402 (3)
C4—C5	1.369 (4)	C13—N1	1.407 (3)
C4—H4	0.930	C14—C15	1.382 (3)
C5—C6	1.394 (3)	C14—H14	0.930
C5—H5	0.930	C15—C16	1.376 (3)
C6—H6	0.930	C15—H15	0.930
C7—C12	1.372 (3)	C16—C17	1.385 (4)
C7—C8	1.375 (3)	C16—C19	1.456 (3)
C7—N1	1.421 (3)	C17—C18	1.381 (3)
C8—C9	1.392 (4)	C17—H17	0.930
C8—H8	0.930	C18—H18	0.930
C9—C10	1.373 (4)	C19—O1	1.200 (3)
C9—H9	0.930	C19—H19	0.930
			
C2—C1—C6	119.9 (2)	C10—C11—C12	120.2 (3)
C2—C1—N1	120.2 (2)	C10—C11—H11	119.9
C6—C1—N1	119.9 (2)	C12—C11—H11	119.9
C1—C2—C3	120.2 (2)	C7—C12—C11	121.1 (3)
C1—C2—H2	119.9	C7—C12—H12	119.5
C3—C2—H2	119.9	C11—C12—H12	119.5
C4—C3—C2	119.9 (3)	C14—C13—C18	118.4 (2)
C4—C3—H3	120.0	C14—C13—N1	121.4 (2)
C2—C3—H3	120.0	C18—C13—N1	120.2 (2)
C3—C4—C5	120.2 (2)	C15—C14—C13	120.4 (2)
C3—C4—H4	119.9	C15—C14—H14	119.8
C5—C4—H4	119.9	C13—C14—H14	119.8
C4—C5—C6	120.3 (3)	C16—C15—C14	121.4 (3)
C4—C5—H5	119.9	C16—C15—H15	119.3
C6—C5—H5	119.9	C14—C15—H15	119.3
C1—C6—C5	119.5 (2)	C15—C16—C17	118.6 (2)
C1—C6—H6	120.3	C15—C16—C19	120.0 (3)
C5—C6—H6	120.3	C17—C16—C19	121.3 (2)
C12—C7—C8	119.0 (3)	C18—C17—C16	120.7 (2)
C12—C7—N1	120.6 (2)	C18—C17—H17	119.6
C8—C7—N1	120.4 (2)	C16—C17—H17	119.6
C7—C8—C9	119.8 (3)	C17—C18—C13	120.4 (3)
C7—C8—H8	120.1	C17—C18—H18	119.8
C9—C8—H8	120.1	C13—C18—H18	119.8
C10—C9—C8	120.2 (3)	O1—C19—C16	126.9 (3)
C10—C9—H9	119.9	O1—C19—H19	116.5
C8—C9—H9	119.9	C16—C19—H19	116.5
C11—C10—C9	119.7 (3)	C13—N1—C7	121.32 (18)
C11—C10—H10	120.1	C13—N1—C1	119.4 (2)
C9—C10—H10	120.1	C7—N1—C1	118.55 (18)
			
C6—C1—C2—C3	−1.3 (4)	C14—C15—C16—C19	−179.6 (3)
N1—C1—C2—C3	−179.5 (2)	C15—C16—C17—C18	0.5 (4)
C1—C2—C3—C4	0.7 (4)	C19—C16—C17—C18	178.7 (2)
C2—C3—C4—C5	−0.2 (4)	C16—C17—C18—C13	0.3 (4)
C3—C4—C5—C6	0.1 (4)	C14—C13—C18—C17	−0.2 (4)
C2—C1—C6—C5	1.2 (4)	N1—C13—C18—C17	−179.6 (2)
N1—C1—C6—C5	179.5 (2)	C15—C16—C19—O1	−177.5 (3)
C4—C5—C6—C1	−0.7 (4)	C17—C16—C19—O1	4.4 (5)
C12—C7—C8—C9	−0.1 (4)	C14—C13—N1—C7	146.1 (2)
N1—C7—C8—C9	177.9 (2)	C18—C13—N1—C7	−34.5 (4)
C7—C8—C9—C10	−0.2 (4)	C14—C13—N1—C1	−23.9 (4)
C8—C9—C10—C11	0.5 (4)	C18—C13—N1—C1	155.4 (2)
C9—C10—C11—C12	−0.5 (5)	C12—C7—N1—C13	−42.9 (4)
C8—C7—C12—C11	0.1 (4)	C8—C7—N1—C13	139.1 (2)
N1—C7—C12—C11	−177.9 (2)	C12—C7—N1—C1	127.3 (2)
C10—C11—C12—C7	0.2 (4)	C8—C7—N1—C1	−50.7 (3)
C18—C13—C14—C15	−0.6 (4)	C2—C1—N1—C13	−58.8 (3)
N1—C13—C14—C15	178.8 (2)	C6—C1—N1—C13	123.0 (3)
C13—C14—C15—C16	1.4 (4)	C2—C1—N1—C7	130.9 (3)
C14—C15—C16—C17	−1.4 (4)	C6—C1—N1—C7	−47.4 (3)
